# Structure of human carbamoyl phosphate synthetase: deciphering the on/off switch of human ureagenesis

**DOI:** 10.1038/srep16950

**Published:** 2015-11-23

**Authors:** Sergio de Cima, Luis M. Polo, Carmen Díez-Fernández, Ana I. Martínez, Javier Cervera, Ignacio Fita, Vicente Rubio

**Affiliations:** 1 Instituto de Biomedicina de Valencia del Consejo Superior de Investigaciones Científicas (IBV-CSIC), Jaime Roig 11, 46010 Valencia, Spain; 2 Group 739, Centro de Investigación Biomédica en Red Sobre Enfermedades Raras del Instituto de Salud Carlos III (CIBERER-ISCIII), Jaime Roig 11, 46010 Valencia, Spain; 3 Centro de Investigación Príncipe Felipe, Eduardo Primo Yúfera 3, 46012 Valencia, Spain; 4 Instituto de Biología Molecular de Barcelona del Consejo Superior de Investigaciones Científicas (IBMB-CSIC), Parc Científic de Barcelona, Baldiri Reixac 10, 08028 Barcelona, Spain

## Abstract

Human carbamoyl phosphate synthetase (CPS1), a 1500-residue multidomain enzyme, catalyzes the first step of ammonia detoxification to urea requiring N-acetyl-L-glutamate (NAG) as essential activator to prevent ammonia/amino acids depletion. Here we present the crystal structures of CPS1 in the absence and in the presence of NAG, clarifying the on/off-switching of the urea cycle by NAG. By binding at the C-terminal domain of CPS1, NAG triggers long-range conformational changes affecting the two distant phosphorylation domains. These changes, concerted with the binding of nucleotides, result in a dramatic remodeling that stabilizes the catalytically competent conformation and the building of the ~35 Å-long tunnel that allows migration of the carbamate intermediate from its site of formation to the second phosphorylation site, where carbamoyl phosphate is produced. These structures allow rationalizing the effects of mutations found in patients with CPS1 deficiency (presenting hyperammonemia, mental retardation and even death), as exemplified here for some mutations.

CPS1 activation by NAG^1^,^2^ has paramount importance, as shown by human NAG synthase deficiency^3^,^4^, which is indistinguishable clinically from CPS1 deficiency but which is successfully treated by giving to the patient the NAG analog and orphan drug N-carbamoyl-L-glutamate^3^,^4^. CPS1 is a true switch operated by NAG levels, which is *on* when the NAG level is high, with highly active ammonia detoxification, whereas when the NAG level is low (*off* situation) CPS1 is less active, decreasing urea cycle operation^5^. Given the high *K*_*m*_ for glutamate of NAG synthase^6^ and the short half-life of NAG *in vivo*^7^, NAG levels reflect the nitrogenous burden manifested in the glutamate level. In this way, ammonia detoxification stops before too low ammonia levels cause depletion of ammonia-derived amino acids such as glutamate and glutamine and the turning on of undue protein catabolism^8^.

CPS1 is a complex multidomain enzyme^9^,^10^,^11^,^12^,^13^ having two separate active centers^13^,^14^,^15^ that catalyze a three-step reaction involving two highly unstable intraenzymatic intermediates^16^,^17^ and intermediate migration between both active centers^13^ (Fig. 1a). Bicarbonate is phosphorylated by one ATP molecule (ATP_A_) to give carboxyphosphate, which is then attacked by ammonia, yielding carbamate, which is phosphorylated by another ATP molecule (ATP_B_) to produce carbamoyl phosphate (Fig. 1a)^13^,^14^,^15^,^16^,^17^. Both phosphorylation steps occur at different sites^13^,^14^,^15^ and depend on NAG^2^,^16^,^18^,^19^. This effector exemplifies extreme allosteric activation, since in its absence CPS1 exhibits ≤2% of the activity at NAG saturation^2^,^20^, largely because NAG increases the *V*_*max*_ and decreases the *K*_*m*_^*ATP*^ of CPS1^20^. Conversely, ATP strongly increases the affinity of CPS1 for NAG^21^. Thus, there is cross-talk between the NAG and nucleotide sites of CPS1, with NAG and ATP bindings being mutually favoured. In addition, CPS1 affinities for its essential ionic activators potassium and magnesium are increased importantly by NAG^20^.

We clarify here the structural mechanism of CPS1 activation by NAG, by determining crystal structures of human CPS1 in NAG-free form (***apo*** form) at 2.4 Å resolution and in NAG and ADP complexed form (***ligand-bound*** form) at 2.6 Å resolution. These two forms represent or approximate the inactive and the active forms of the enzyme, respectively. Their comparison has revealed important conformational changes that involve the NAG binding site in the C-terminal region of the enzyme and the two nucleotide binding sites that are more than ~35 Å away, providing structural clues on CPS1 regulation and functioning. Furthermore, as exemplified here with a few CPS1 mutations, these structures set the frame for rationalizing the effects of the >130 clinical single amino acid substitutions reported in patients with CPS1 deficiency (CPS1D) that are spread over the entire CPS1 molecule^22^. Our structures might also help understand how the reported acylations of some CPS1 lysines and their deacylation by Sirt5 could control urea cycle activity, linking CPS1 activity control with aging processes and with the hyperammonemia of glutaric aciduria^23^,^24^,^25^,^26^.

## Results and Discussion

### Overall human CPS1 architecture

The crystals of the *apo* and the *ligand-bound* forms of human recombinant CPS1^27^ (lacking the N-terminal mitochondrial targeting peptide, residues 1–37; replaced by a His-tag to facilitate purification) contained in their asymmetric units (Table 1), one or two similar dimers (Supplementary Fig. 1), respectively. These dimers resulted from contacts involving <3% of the molecular surface (1300 or 1100 Å^2^ per subunit in the *apo* and *ligand-bound* forms, respectively), indicating poor dimer stability (PISA server, http://www.ebi.ac.uk/pdbe/pisa)^28^, consistent with the behavior of human CPS1 in solution as a monomer-dimer system in rapid equilibrium in which the monomer predominates^2^,^27^,^29^. Dimerization, mediated by exposed parts of the C-terminal domain and of the end of the preceding domain, is not analyzed here any further, since CPS1 monomers and dimers are active when NAG is present^30^.

**Figure 1:**
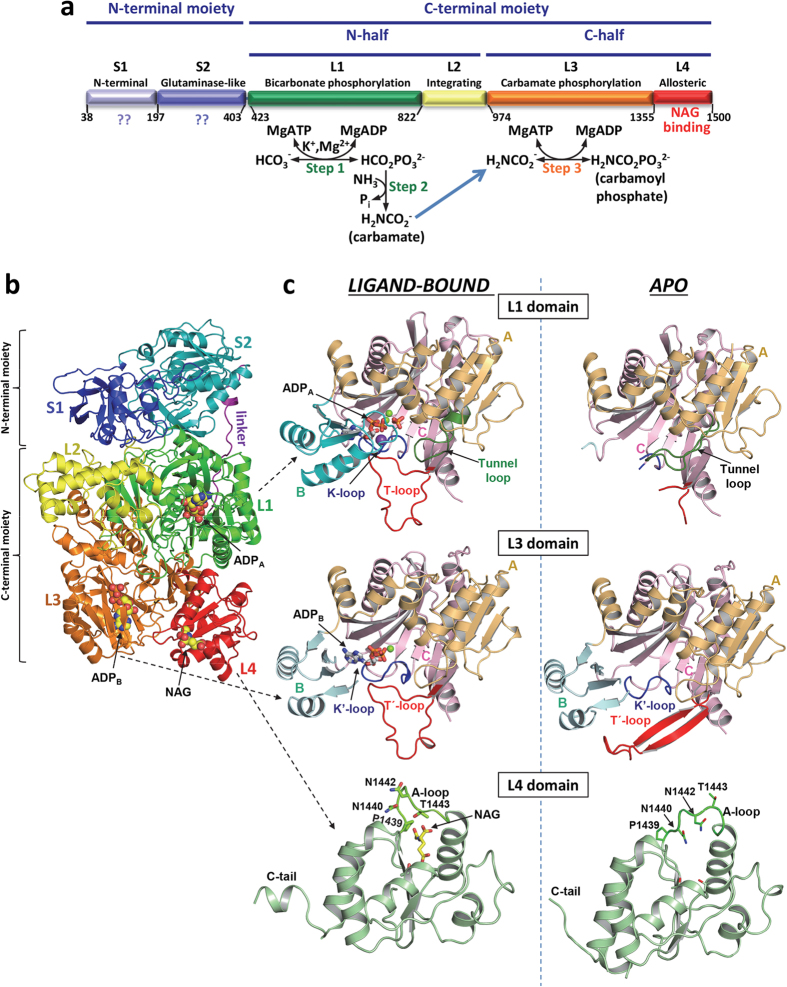
Structure of human CPS1. (**a**) Scheme of the mature CPS1 polypeptide, indicating its two moieties (top), which are homologous to the small and large subunits of CPS from *E. coli.* Also indicated are the two sequence-related halves of the large moiety (middle). Domain composition with names and boundaries, given as residue numbers, is indicated in the lower bar. The three steps of the CPS1 reaction are shown below the corresponding domains where they occur. The blue arrow indicates carbamate migration between the phosphorylation active centers, a process contributed mainly by residues from both phosphorylation domains. (**b**) Cartoon representation of the CPS1 monomer, with domains shown in different colors, and labeled. The structure depicted corresponds to the *ligand-bound* conformation. The two ADPs and NAG molecules found bound to CPS1 are shown in space-filling representation. In (**c**), domains L1, L3 and L4 are shown expanded, in the *ligand-bound* (left) and *apo* (right) forms. In L1 and L3 the three subdomains A, B and C, and important loops mentioned in the text are labeled and represented with different colors. In the L4 domain some residues from the A-loop are shown (as sticks) to highlight the large structural changes of these side chains when NAG is bound. The ADP molecules, a phosphate and NAG are shown in rods representation, magnesium ions are shown as green spheres and the potassium ion bound to the K-loop is shown as a violet sphere.

The CPS1 monomer presents the previously inferred^9^,^10^,^11^,^12^,^13^ 6-globular domain architecture (Fig. 1a,b and Supplementary Fig. 2), with 2-domain and 4-domain respective N- and C-terminal moieties that correspond, respectively, to the small and large subunits of bacterial CPSs (these are heterodimeric, intrinsically active and NAG-independent CPSs)^31^,^32^, with an extensive interface of ~2400 Å^2^ between these moieties. An extended 20-amino acid ~50 Å linker, well conserved among the CPS1 family, which connects these moieties (Fig. 1b), appears quite mobile (up to 7 Å displacements in its central region observed in different CPS1 subunits).

Of the S1 and S2 domains that compose the N-terminal moiety, S2 is an ancestral but inactive^2^,^9^ glutamine amidotransferase/glutaminase-like domain. The four domains that compose the C-terminal moiety include two homologous catalytic domains^9^,^13^, L1 and L3, which phosphorylate respectively bicarbonate and carbamate^13^. L1 and L3 are linked by an intervening domain (L2) recently defined as an integrating domain^33^, which exhibits a unique fold of seven α-helices and two β-strands (Fig. 1b). The C-terminal or allosteric domain L4 presents a methylglyoxal synthase-like fold^34^ and was shown to bind NAG^35^. Helices, strands and loops will be named here as shown in Supplementary Fig. 2, giving the domain (S1, S2, etc.) and then the sequential numbering of the secondary structure element within the domain (S2α1, L4β2, etc.).

Although exhibiting the same overall architecture (averaged r.m.s.d., 1.3 Å for the superposition of 1285 Cα atoms), the *apo* and *ligand-bound* forms present large differences (Supplementary Figs 2 and 3) that arise from structural rearrangements in domain L4 and in both phosphorylation domains L1 and L3 (Fig. 1c and Supplementary Fig. 3). The differences also reflect reorientations of domains (Supplementary Video 1), particularly of domain L4 with respect to domains L1 and L3, with respective rotations of 13° and 9.3° (Supplementary Table 1) and with changes in the buried surface areas between these domains (Supplementary Table 2).

### NAG binding and signal transmission to the phosphorylation domains

**Figure 2:**
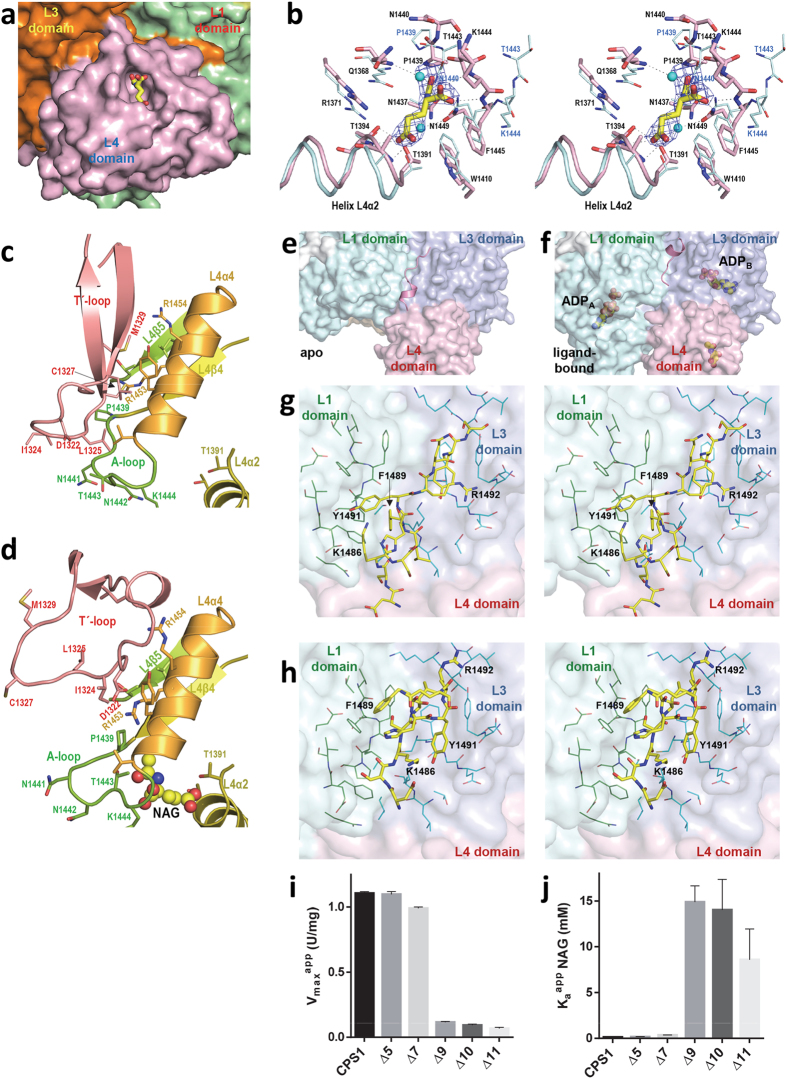
The allosteric L4 domain. (**a**) View of NAG bound in its binding pocket in the L4 domain, not far from the interface with the L3 domain. (**b**) Stereo view of the superposition of the NAG site of the *apo* (carbon atoms colored cyan) and *ligand-bound* (protein and NAG carbon atoms colored pink and yellow, respectively) forms, identifying the residues by labeling them. When the position of one residue changes much, the residue is labeled in blue for the *apo* form. The unbiased (F_o_-F_c_) omit electron density map obtained prior to building the ligand is shown as a blue grid contoured at 2σ. (**c**,**d**) Helix L4α4 and some adjacent regions of the L4 domain and the T′-loop from domain L3 are shown, in the (**c**) *apo* and (**d**) *ligand-bound* forms. NAG is shown in spheres representation. The side-chains of some residues are shown in sticks representation and some secondary structure elements and residues are identified by labeling in the same color as the structural element to which they belong. (**e**,**f**) C-tail from domain L4 interacting with neighbor domains L1 and L3, in the *apo* and *ligand-bound* forms, and (**g**,**h**) respective close-up stereo-views of (**e**,**f**) with some residues labeled to highlight the radical changes of both the C-tail structure and their interactions with neighbor domains. (**i**,**j**) Influence of C-terminal truncations of the indicated length (Δn denotes the number of deleted residues starting from the C-terminus) on (**g**) the activity of the enzyme at saturation of NAG (*V*_*max*_^*app*^), and on (**h**) the concentration of NAG (*K*_*a*_^*app*^*NAG*) needed for attaining an activity of 0.5 × *V*_*max*_^*app*^. CPS1 denotes the wild-type enzyme.

One NAG molecule was found sitting with full occupancy in each subunit of *ligand-bound* CPS1 (Supplementary Fig. 1), in a pocket of domain L4 that is close to the interface with domain L3 (Fig. 2a). The NAG molecule utilizes its three C^α^ substituents for stereospecific anchoring on the enzyme (Fig. 2b). These findings agree with the results of solution studies revealing the binding of one NAG molecule per enzyme subunit^21^, of structure-reactivity investigations on the requirements of the ligand for binding at the NAG site of CPS1^36^, and with the exclusive photoaffinity labeling of domain L4 with the NAG analogue N-chloroacetyl-L-glutamate^35^. Thus, NAG binding to CPS1 as observed in the crystal structure appears to genuinely reflect the binding of this effector to the enzyme *in vivo*.

Of the three non-hydrogen substituents of the C^α^ of NAG, the α-COO^−^ interacts extensively with the encircling L4β4-L4α4 loop (**A-loop**; residues 1439–1445; Fig. 1c, bottom), forming hydrogen bonds with backbone nitrogen atoms (Gln1368, Lys1444, Phe1445) and with the Asn1449 side chain. The acetamide moiety of NAG is hydrogen-bonded via its O atom with the -OH of Thr1443 and inserts its methyl group in a sub-pocket formed by Asn1437, Pro1439 and Asn1449. Only minor adjustments in this sub-pocket are needed for accepting the amido group of the NAG analog N-carbamyl-L-glutamate used therapeutically in NAG synthase deficiency^3^,^4^. The third substituent of the NAG C^α^ makes hydrophobic contacts with Gln1368 and Trp1410 and faces with its δ-COO^−^ the N-end of helix L4α2 (residues 1391–1400), forming hydrogen bonds with this helix via backbone atoms (Ala1393 and Thr1394) and side chains atoms (Thr1391 and Thr1394) (Fig. 2b). These interactions account for the observations^36^ that all alterations tested in the three substituents of the NAG C^α^ reduced the affinity for CPS1, excepting the addition of a phenyl group to the C^β^ of NAG, which was without substantial effect. This lack of effect is explained by the present structure, given the exposure of the face of the C^β^ of NAG, where the phenyl substituent would be found, and the nearness of the hydrophobic chains of Trp1410 and Phe1445 (Fig. 2b), which could interact with the phenyl group of the modified ligand.

Despite the triggering by NAG of extreme allosteric activation, domain L4 does not exhibit large structural differences when the *apo* and the *ligand-bound* crystals are compared (r.m.s.d., 1.1 Å for 124 superposed Cα atoms). Only three sites of this domain exhibit significant differences (Supplementary Fig. 3). One such site is the hinge (Gly1354-Pro1358) for reorientation of the L4 domain relative to domain L3, at the connection between these domains (Supplementary Video 1). In the NAG-interacting A-loop, the seven residues forming this loop alter their conformation (Fig. 1c, bottom panel and Fig. 2c,d), with movements of up to 9 Å of Asn1440 and Thr1443. The **C-tail** (residues 1481–1500) (Supplementary Fig. 3) that projects away from domain L4 and sits along a groove between both phosphorylation domains, closely interacting with them (Fig. 2e-h), forms a short helix in the *ligand-bound* form that is absent in the *apo* form. This change is associated with drastically different C-tail interactions with neighbour domains in both crystal forms (Fig. 2g,h and Supplementary Video 1). Given the remoteness from the bound NAG (Fig. 1c, bottom panels), these C-tail differences do not result from direct interactions of the tail with the activator. However, limited proteolysis studies had shown that these last 20 residues of rat CPS1 are extremely important for activation, with their deletion causing a >30 fold increase in the *K*_*a*_^NAG^ (the NAG concentration needed for half-maximal activation) and in the *K*_*m*_^ATP 11^. Human CPS1 variants with progressively shorter C-ends show that the *K*_*a*_^NAG^ is abruptly increased (thus, the apparent affinity for NAG is decreased) and the apparent *V*_*max*_ is importantly decreased when the last nine residues are eliminated (Fig. 2i,j), confirming the importance of the C-tail.

L4 domain residues contributing to CPS1 activation by NAG should change due to NAG binding and should be involved in interdomain interactions that transmit the NAG signal to the phosphorylation domains. Excluding the C-tail, only three residues from the L4 domain, Thr1443 and the pair Pro1439/Arg1453, fulfill these requirements (Fig. 2c,d). In the *ligand-bound* form, Thr1443 interacts with NAG, while in the *apo* form it interacts with Leu1325 from the L3 domain (Fig. 2b–d). Changes in Pro1439 associated with NAG binding (Figs 1c and 2b–d) affect Arg1453, which makes a salt bridge with Asp1322 and interacts weakly with Ile1324 (both from the L3 domain) in the *ligand-bound* form, while in the *apo* form Arg1453 interacts with Cys1327 and Met1329 (Fig. 2c,d). All the residues of the L3 domain mediating these interactions belong to the L3β15-L3β16 loop (residues 1311–1333), named here the **T′-loop** (from signal transmission loop), which transitions from a β-hairpin in the *apo* form to a 20 Å-wide expanded loop in the *ligand-bound* form (Fig. 1c, middle panels, Fig. 2c,d and Supplementary Figs 2 and 3). The changing interactions of Thr1443 and Arg1453 with the T′-loop appear to stabilize one or the other form of this loop in parallel with whether NAG is or is not bound, possibly being essential for signal transmission in the NAG-operated switch. Indeed, the finding^37^ in patients with severe NAGS deficiency of the mutations Pro1439Leu, Thr1443Ala, Arg1453Gln and Arg1453Trp, emphasize the key role played by these three L4 domain residues. Furthermore, the introduction in CPS1 of the Asp1322Leu, Arg1453Gln and Arg1453Trp mutations caused enzyme inactivation without apparent impairment of enzyme folding^37^,^38^, as expected if NAG activation were abolished by preventing the transmission of the activating signal from L4 to the remainder of the enzyme.

Superposing the *apo* conformation of domain L4 (excluding the C-tail) onto the *ligand-bound* conformation of CPS1 results in Arg1453 clashing with Ile1324, which should destabilize the *apo* conformation of Pro1439. This agrees with the enhancement of NAG binding via the stabilization of the active conformation of CPS1, as exemplified by using cryoprotectants, which are NAG-independent activators of CPS1^2^,^39^. Superposing the ligand-bound conformation of domain L4 (excluding the C-tail) onto the *apo* conformation of CPS1 results in only a minor contact of Asn1441 with Phe1143 and in some contacts of Arg1453 mainly with Cys1327. Therefore, NAG binding to domain L4 contributes to trigger structural changes towards the active conformation in neighbor domain L3, though the reorganization required affects very few residues.

### Changes in the phosphorylation domains and in nucleotide binding

As expected from the mutual cross-talk between NAG binding and nucleotide binding in CPS1^20^,^21^, the active form of the enzyme represented by the *ligand-bound* form not only has NAG bound in each monomer, but also binds two molecules of the ADP added in the crystallization mixture (Fig. 1b and Supplementary Fig. 1). Each ADP sits in one phosphorylation domain (L1 and L3 domains, Fig. 1c), with the adenine ring sandwiched between the central β sheets of the B and C subdomains (for the boundaries between the A, B and C subdomains, see Supplementary Fig. 2), as is characteristic for the ATP-grasp fold^40^. Also as it might have been expected for domains having arisen from an ancestral duplication^9^, the two phosphorylation domains form a pseudohomodimer (Fig. 1b) with nearly exact twofold pseudosymmetry (174° and 178.5° rotations and r.m.s.d. values of 2.1 and 1.8 Å for superposition of 269 and 330 Cα atoms in the *apo* and *ligand-bound* forms, respectively).

**Figure 3:**
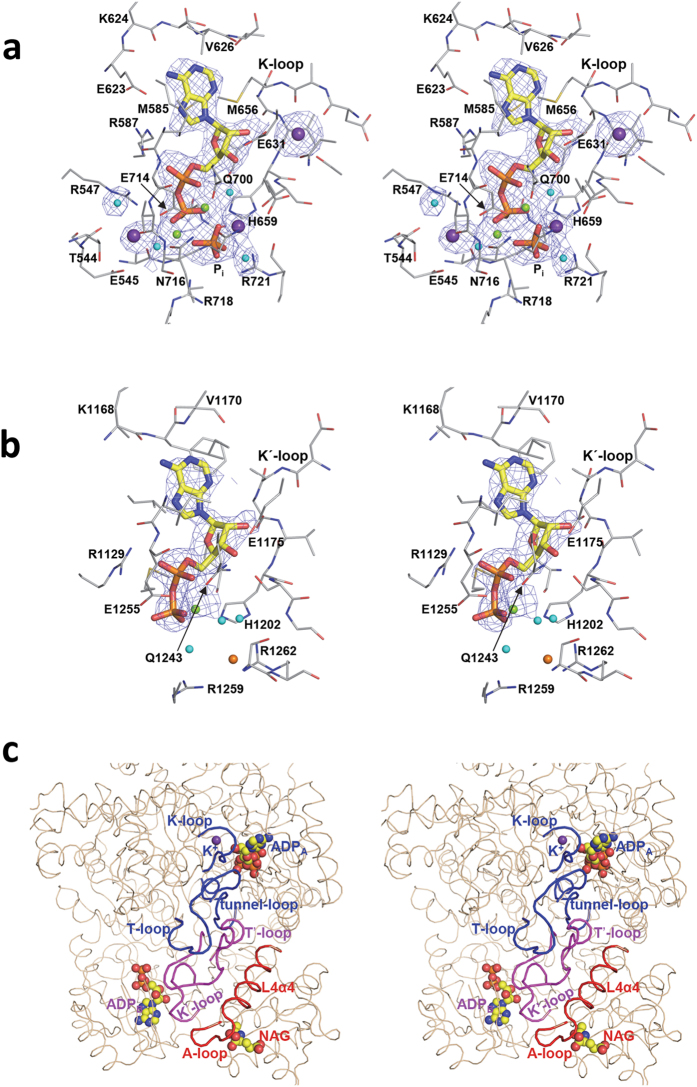
Stereo views of the binding sites for the two molecules of the nucleotide that are used in the reaction, and for the loop system used in signal transmission. ADP binding in domains L1 (**a**) and L3 (**b**). Protein and ADP and phosphate are shown in sticks representation (carbon atoms in grey for the protein and in yellow for the ligands). The lone phosphate is labeled P_i_. Potassium, magnesium, chloride ions and water molecules are spheres colored purple, green, orange and cyan, respectively. The (F_o_-F_c_) electron density maps, computed without the ligands, are shown as a blue grid contoured at 2σ. (**c**) Close up of the structure of the majority of the C-terminal moiety, to show the binding of both ADP molecules and of NAG and to highlight the relations of helix L4α4 and of the loops more centrally involved in signal transmission. Whereas the overall structure is in brown thin string, the highlighted elements are in thicker string and are labeled, with red, magenta and blue coloring depending on whether they belong to L4, L3 or L1, respectively.

Although being homologous and carrying out phosphorylations of the analogous compounds bicarbonate and carbamate (Fig. 1a), the catalytic properties of both phosphorylation domains L1 and L3 are not identical^41^. These differences manifest themselves in the present structures. The structures of the L3 domain are highly similar in the *apo* and *ligand-bound* forms (r.m.s.d., 0.83 Å for 340 residues). Virtually all elements of this domain are observed in both crystal forms (Fig. 1c, middle panels). In contrast, in the case of the L1 domain there are ~90 residues that are disordered (and thus that are not seen) in the *apo* form and that become ordered when the enzyme has the ligands bound to it (Fig. 1c, top panels). These structural findings parallel the observations that ATP_B_ binds to domain L3 irrespective of whether NAG is or is not bound^20^,^42^, whereas the affinity of the enzyme for ATP_A_, the ATP molecule that binds in domain L1, is extremely low in the absence of NAG^20^. Indeed, the crystal structures account for this decreased affinity, since in the *apo* form, (Fig. 1c, top right panel) the entire B subdomain of domain L1, and loop L1β11-L1β12 (residues 654–662; called here the **K-loop**) could not be traced, indicating that they are mobile or disordered. In contrast, in the *ligand-bound* form (Fig. 1c, top left panel) the entire B subdomain is well traced in this domain, closing on the bound ADP, with loop L1β11-L1β12 being fully structured, having at its center a coordinated potassium ion (thus the name K-loop) and being involved in intimate interactions with the bound ADP (Fig. 3a,c). Interestingly, the L1β17-L1β18 loop (residues 777–793; named here the **T-loop** because of its equivalence in the L1 domain to the T'-loop of the L3 domain) could not be traced in the *apo* form. This loop became fixed and structured in the *ligand-bound* form (Fig. 1c, top panels), presenting the same wide open loop shape as the T'-loop (compare with Fig. 1c, middle left panel). This structural fixation appears largely due to the interactions of this loop with the widened T′-loop (Fig. 3c). Indeed, the T'-loop was the only part of the L3 domain that exhibited important conformational differences between the *apo* and the *ligand-bound* forms (Fig. 1c, middle panels and Supplementary Figs 2 and 3).

**Figure 4:**
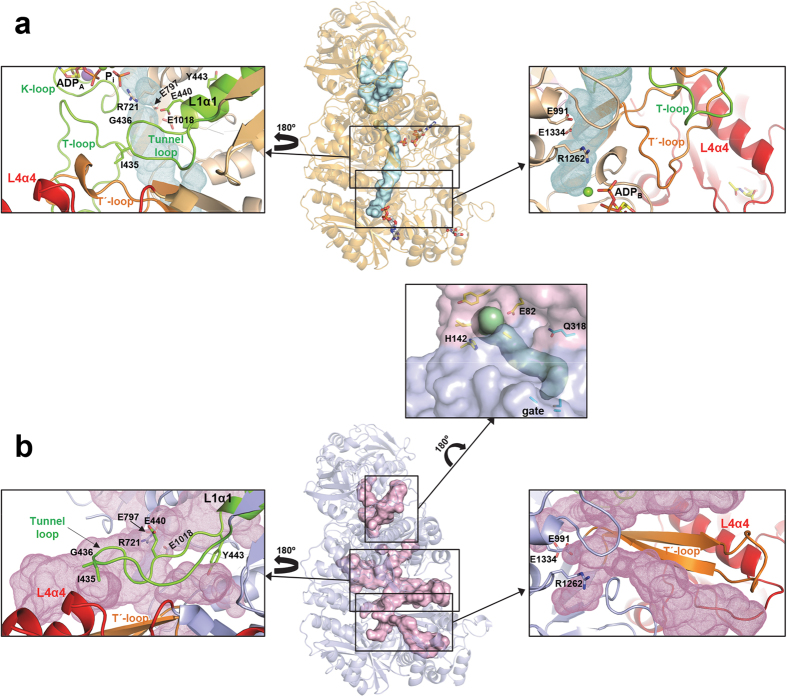
Tunnels and cavities in CPS1. Overall views (center) of tunnels and cavities (surface representation) in (**a**) the *ligand-bound* and (**b**) the *apo* forms of CPS1. The carbamate tunnel linking the two phosphorylation active sites (see text) is well defined only in the *ligand-bound* conformation. Boxes at both sides show detailed views of the structural elements forming the carbamate tunnel. The tunnel loop (colored green) and the T′-loop (colored orange) occlude in the *ligand-bound* form many of the cavities found in the *apo* form. Some side chains are explicitly shown to highlight the structural changes. A possible ammonia tunnel, which remains invariant between the *apo* and ligand bound forms, is depicted in the central box. Residues at the entrance to this tunnel and at the conserved gate are shown with carbon atoms colored yellow and cyan, respectively.

The two phosphorylation steps of the CPS1 reaction also differ in that the step of bicarbonate phosphorylation requires potassium and excess magnesium over that for complexing the nucleotide, whereas these needs are not patent for the carbamate phosphorylation step (Fig. 1a, reactions)^19^,^41^. In agreement with these differences, in the *ligand-bound* form the L3 domain had no potassium complexed to its K'-loop and it contained only the magnesium ion that is complexed to the ADP, whereas the L1 domain besides having also two bound magnesium ions, hosted a potassium ion coordinated to its K-loop (Figs 1c and 3a,b). These two magnesium ions are coordinated to both the ADP molecule and to one nearby phosphate molecule (Fig. 3a) that, given its location relative to the ADP, may represent the phosphate moiety of the carboxyphosphate intermediate made at this phosphorylation site by the attack of bicarbonate on the ATP. Indeed, although no bicarbonate was observed in the structures, the fact that the mutation to Met of the nearby Thr544 (Fig. 3a) found in a patient with CPS1 deficiency^27^ selectively increased ~60-fold the K_m_ for bicarbonate, points to the binding of bicarbonate in the vicinity of the phosphate. In any case, this phosphate interacts with the highly conserved Arg721 (Fig. 3a), possibly reflecting the corresponding binding of this arginine to ATP_A_, increasing the susceptibility of the γ-phosphate to the attack by bicarbonate. This arginine, which in the absence of ligands interacts with Glu440, interacts in the *ligand-bound* form not only with the phosphate but also with Gly436, a residue that is at the tip of the L1β1-L1α1 loop (the **tunnel-loop**, residues 430–443) of subdomain A. This last interaction should help stabilize this loop in the conformation that delineates the carbamate tunnel that characterizes the activated form of the enzyme (Fig. 4a, center; discussed below). It is interesting that Arg1262, which is the residue corresponding in domain L3 to Arg721 of domain L1, interacts with a chloride ion that sits approximately at an equivalent position to that of the phosphate in the L1 domain (Fig. 3b).

**Figure 5:**
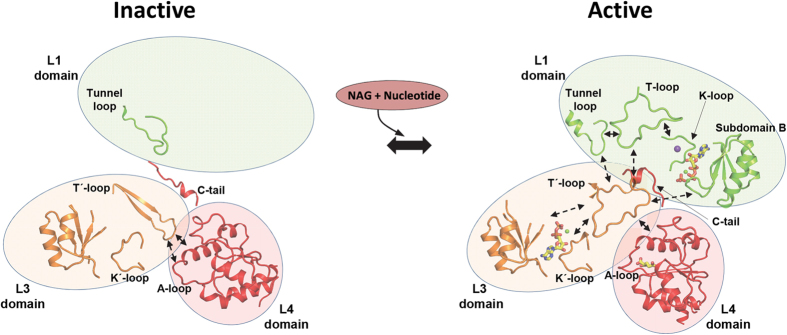
Scheme of CPS1 activation. Schematic representation of the structural changes and interactions (indicated as double dashed arrows) between the different elements involved in the activation of CPS1 upon binding of NAG and the nucleotide. Ligands are shown in sticks representation. Binding of NAG alters the A-loop, which in the *apo* form interacts with the T′-loop from the L3 domain. This loop experiences a radical reorganization between the apo and *ligand-bound* forms. The T′-loop interacts also with the T-loop (from the L1-domain) influencing both ATP binding sites mainly through their interaction with the K′- and K-loops, respectively. These changes together with the remodeling of the tunnel-loop and reorientations between domains result in the building of a functional carbamate tunnel. Stabilization of the modified architecture is achieved through the C-tail fixing the relative orientations of domains L1, L3 and L4.

As already discussed, the L3 domain, and in particular the T′-loop, can play an important role in allosteric activation due to both its extensive interdomain interactions (Fig. 3c) and the already commented important changes between the *apo* and *ligand-bound* forms (Fig. 1c, middle panels, Figs 2c,d and 4, right boxes, and Fig. 5). Thus, the ~20 Å-wide loop observed in the *ligand-bound* form interacts not only with helix L4α4 (to which Arg1453 belongs), but also with the T-loop, the tunnel-loop and subdomain B from domain L1, and with the K′-loop from domain L3, indicating that this T′-loop can be a hub for NAG signal transmission to the catalytic machinery (Fig. 3c).

### The carbamate tunnel is aberrant in the *apo* form and becomes functional in the *ligand-bound* form

Carbamate, a highly labile compound produced in domain L1 by the reaction between carboxyphosphate and ammonia, has to be transported intramolecularly ~35 Å, shielded from water, to the active center of domain L3, where it is phosphorylated to give carbamoyl phosphate (Fig. 1a)^13^. This transport can take place through a tunnel connecting the active centers of domains L1 and L3, similarly to what was proposed for *Escherichia coli* CPS^32^,^34^,^43^ (EcCPS). In CPS1 the possible carbamate tunnel, roughly linear and with a diameter of 2.6–5 Å, is well defined only in the *ligand-bound* form (Fig. 4a), where it is formed by residues evenly belonging to domains L1 and L3, according to the quasi-two fold symmetry that relates these two domains. At each end of the tunnel Arg721 and Arg1262 can participate, as indicated before, in the binding to the nucleotide γ-phosphate in domains L1 and L3, respectively. These arginine residues, together with five conserved glutamic acid residues at the beginning (glutamates 440, 797 and 1018) and at the end (glutamates 991 and 1334) of the tunnel, can play a role in the entry and exit of carbamate to and from the tunnel^43^,^44^,^45^.

In the *apo* form of CPS1 cavities and tunnels increase and change dramatically with respect to the ones seen in the *ligand-bound* form (Fig. 4a,b), with several expansions, in particular in the central part of the tunnel, and three large lateral branches that open at the molecular surface (Fig. 4b). Two of these branches are almost perpendicular to the direction joining the phosphorylation centers, with one branch parallel to the tunnel-loop helix L1α1 (Fig. 4b, left box). In the *ligand-bound* form these two branches are occluded mainly by the lengthening by 1.5 turns of helix L1α1 and by the related remodeling of the tunnel-loop. This loop, placed between domains L1, L3 and L4, shrinks and establishes new hydrogen bonds mainly with loop L3β2-L3β3 (residues 1014–1027; only Glu1018 is seen in Fig. 4, left boxes) and with the T′-loop (Figs 3c and 4a left box and Fig. 5). Similarly, a third lateral branch is occluded in the *ligand-bound* form by, in particular, the remodeling of the T′-loop (Fig. 4, right boxes). Formation of a functional carbamate tunnel in the active-like *ligand-bound* form appears to indicate that the main restraint for the large cavities and tunnels found in the *apo* form is to allow the structural rearrangements required for CPS1 activation. In summary, the structural changes that lead to definition of the appropriate carbamate tunnel in the *ligand-bound* form appear to be key elements in the process of CPS1 activation.

### An incomplete catalytic triad for glutamine use, and a potential ammonia tunnel.

Glutamine is utilized as the endogenous source of ammonia by all types of CPS^31^,^46^,^47^,^48^ except CPS1^2^. The inability of human CPS1 to use glutamine is explained by the replacement by serine (Ser294) in this enzyme of the key catalytic cysteine of the ***catalytic triad*** of histidine, glutamate and cysteine^34^,^49^ used for glutamine hydrolysis by other CPSs (Supplementary Fig. 4). However, other factors might contribute also to the lack of glutaminase activity of human CPS1, since *Rana catesbeiana* CPS1 conserves the catalytic triad but is unable to utilize glutamine, recovering the ancestral glutaminase activity by mutating to leucine a lysine that corresponds to Thr295 of human CPS1^50^. In any case, CPS1 must take ammonia from the environment and should deliver it to the carboxyphosphate production site at the beginning of the carbamate tunnel. Cavities and tunnels observed in the CPS1 N-terminal moiety in both the *apo* and *ligand-bound* forms (Fig. 4), are likely related with this ammonia intake and delivery. Although CPS1 maintains many elements of the EcCPS ammonia tunnel going from the ancestral glutaminase site to the conserved gate (CPS1 residues Cys648, Ala667 and Ala729) at the end of this tunnel^51^ in domain L1, Gln318 replaces Gly293 of EcCPS and blocks this tunnel before the gate. However, there is an alternative potential path for intake of external ammonia. This path could correspond to the tunnel that skips the ancestral glutaminase active site of CPS1, starting at the molecular surface, between residues Glu82 and His142, and continuing parallel to the interface between the N- and C-terminal moieties, reaching the gate of the carbamate tunnel (Fig. 4b, box at the center). Charged residues at the entrance of this alternative tunnel might facilitate the entry of uncharged ammonia, the true CPS1 substrate^14^, rather than of NH_4_^+^.

### The CPS1 structure allows rationalizing the effects of clinical mutations

The large number of "private" missense mutations with little recurrence found in individual patients with CPS1 deficiency^22^ (Supplementary Figs 2 and 5) and the lack in most cases of appropriate pedigree information, render crucial to clarify whether a given mutation is disease-causing or is merely a rare unreported trivial polymorphism. The present structures are valuable to rationalize the consequences of individual mutations, as exemplified here with the reported clinical mutations that affect the L4 domain^22^ (Supplementary Fig. 5b). These mutations appear a good sampling set since their effects were examined experimentally using recombinant CPS1^27^,^37^,^38^. In the crystal structure, the Arg1371Leu, Thr1391Met and Thr1443Met mutations eliminate key polar interactions with NAG (Fig. 2b and Supplementary Fig. 5b), agreeing with the observations that these mutations increase two orders of magnitude the *K*_*a*_^NAG^ 27,37. As indicated before, the key role in signal transmission of the Arg1453/Asp1322 ion pair (Fig. 2d) predicts abolition of NAG activation by the Arg1453Trp and Arg1453Gln clinical mutations, again agreeing with the experimental observation that these mutants were inactive^38^. By altering the A-loop of the NAG site that changes its conformation in the *ligand-bound* form of CPS1 (Fig. 1c, bottom panels; and Fig. 2c,d), the Pro1439Leu mutation may be expected to impair NAG binding, as observed (~15-fold increase in *K*_*a*_ for NAG)^37^. The Pro1462Arg mutation, by drastically affecting a structurally very important residue at the beginning of L4β5, should impair CPS1 activation, given the interaction of L4β5 with the wide form of the T′-loop that is observed in the *ligand-bound* crystal (Supplementary Fig. 5b). Correspondingly, this mutation decreased *V*_*max*_ ~30-fold^37^. In contrast, Pro1411 is in an external loop that is not involved in NAG binding neither in the interactions with other domains or in dimer formation (Supplementary Fig. 5b). Thus, the Pro1411Leu substitution should have little effect, agreeing with its finding in a patient without a drastic disease phenotype^37^, and with the observation that this substitution did not cause important changes in the properties of the *in vitro* expressed enzyme^38^. The mutation Tyr1491His^38^, affecting a C-tail residue, may be predicted to drastically affect the NAG activation process, and, indeed, the affinity for NAG was decreased ~60-fold by this mutation^38^. Even misfolding or destabilizing mutations were amenable to rationalization of their effects. The mutation Leu1381Ser, causing misfolding and degradation, and the Ala1378Thr and Leu1398Val mutations, which decreased the thermal stability of CPS1^27^,^37^, affect the crowded, highly hydrophobic patch that glues the central β sheet and the α_3_ layer in the α_3_β_5_α_2_ sandwich that constitutes the L4 domain fold (Supplementary Fig. 5b). Differences between wild-type and mutant forms in side-chain polarity and/or in size can account for the misfolding or the thermal destabilization observed with these mutations. Overall, it was possible to rationalize the effects of most of the reported clinical mutations that map in this domain, suggesting that similarly accurate rationalizations, and even predictions, could be made concerning the effects of the mutations affecting other domains.

### Final remarks

The present structures clarify the activation mechanism of this intrinsically inactive enzyme. NAG, by binding to domain L4, triggers changes in the A-loop and in Arg1453 (Fig. 1c, bottom panels and Fig. 2c,d) that result in changing interactions with the T′-loop of domain L3, which reorganizes completely from a β-hairpin in the *apo* form to a widened loop in the *ligand-bound* form (Figs 1c and 5). In this last form, the T′-loop interacts also with the tunnel-loop and the T-loop of the L1 domain, thus transferring the activating information to the bicarbonate-phosphorylating domain, whereas inside domain L3 the T′-loop interacts with the K′-loop (Figs 3c and 5). Binding of a nucleotide to domain L3 should cause only limited stabilization of the active conformation, given the small remodeling required, mainly of the K′-loop. In fact, nucleotide binding at this site was reported to be independent of NAG^20^,^42^. The sole binding of either NAG alone or of a nucleotide to domain L3 most likely could not trigger efficient activation. In domain L1, the T-loop interacts with the K-loop, which changes, from disordered in the *apo* form, to establishing strong interactions with the bound nucleotide and with a potassium ion in the *ligand-bound* form (Figs 3c and 5). Therefore, a bound nucleotide in the bicarbonate phosphorylation domain L1 should have a major stabilizing effect on the active conformation, in line with the observations made in NAG binding studies^21^. However, by the same reason, binding of the nucleotide to a not yet active conformation is not favored, again in line with studies of the reaction in the absence of NAG^20^.

The activation process also involves changes in the relative orientations of the L4 domain and the phosphorylation domains. These changes are reflected in ~10° rotation of L1 and L3 (Supplementary Table 1), and the extensive loop remodeling resulting in the delineation of a well-defined carbamate tunnel (Fig. 4) that should enable shielded passage of carbamate from its formation site in domain L1 to its phosphorylation site in domain L3. However, ADP release from domain L1 at the end of each catalytic cycle should destabilize the active conformation. The C-tail may lock the active conformation by providing a stability barrier, preventing the return of the different domains and of the carbamate tunnel to the orientations and shapes observed in inactive CPS1. This locking may explain the lag effects observed both during activation when the enzyme is first exposed to a mixture of NAG and the CPS1 substrates, or during deactivation (even if NAG is lost, CPS1 remains active for some time)^15^,^20^,^52^. C-tail control also agrees with the observation that the last 20 C-terminal residues are important for the cross-talk between activator and phosphorylation sites that characterizes the allosteric activation of CPS1 (Fig. 2i,j)^11^.

The present structures also shed light on why CPS1 cannot use glutamine, given the substitution of the catalytic Cys by a Ser. However, glutamine binding might be impeded by other glutamine binding site alterations, and, in fact, the passage of glutamine-derived ammonia might be prevented by changes in the ancestral ammonia tunnel that connects the remnant of the glutaminase site with the large subunit^32^,^34^. In a mutant of frog CPS1 with artificially gained glutaminase activity, ammonia was excreted, later re-entering through another path, agreeing with the blockage of the ancestral ammonia tunnel^50^, and with the existence of an alternative path for external ammonia intake, that could correspond to the one proposed here (Fig. 4b, central box). A less clearly defined similar alternative tunnel might also be present in EcCPS (PDB 1JDB). Intake of free ammonia through this alternative tunnel would be in agreement with the lack of activity with glutamine as substrate of EcCPS variants in which the tunnel from glutamine was blocked or perforated, since these variants were normally active with external ammonia^44^,^53^,^54^. In contrast, variants altering the gate affected importantly the activity with both glutamine and free ammonia^51^.

Despite the important functional differences with CPS1, the glutamine-dependent, NAG insensitive, CPS from *E. coli*, a heterodimer regulated by ornithine whose active-like structure was determined nearly 20 years ago^32^, has been used for homology modeling of CPS1 in numerous structure-function studies of missense mutations of human CPS1^22^,^27^,^33^,^37^,^38^,^55^. Due to the intricate network of interactions and remodeling found in the structures of CPS1, it is now clear that variants can interfere at the level of substrate binding and catalysis, but also by altering the tunneling of intermediate products or by interfering with the rearrangements required by regulation and activation. Results from this work provide a unique structural framework to interpret the molecular pathologies, as exemplified above, and might also help assess the consequences of the post-translational modifications that are presently being identified in CPS1^23^,^24^,^25^,^26^ some of which appear to be endowed with activity-regulating roles and to be susceptible to removal by the anti-aging protein Sirt5. Therefore, our structural studies may open the gate to further structure-functional studies that might connect the present knowledge to superposed levels of regulation and of biological significance.

## Materials and Methods

### Wild type and mutant enzyme production, purification, site-directed mutagenesis and enzyme activity assay

With the aim to generate enzyme forms lacking 11, 10, 9, 7 and 5 C-terminal residues, stop codons were introduced to replace the CPS1 coding codons at positions (as codon numbers) 1490, 1491, 1492, 1494 and 1496, using site-directed mutagenesis by the overlapping extension method^27^ (Quickchange kit from Stratagene), utilizing appropriate forward and reverse oligonucleotide primers (Supplementary Table 3). We corroborated by sequencing the correctness of the constructs, the presence of the desired codon change and the absence of unwanted mutations.

Either wild-type or mutant human mature CPS1 with an N-terminal His_6_-tag was expressed in a baculovirus/insect cell system and purified as previously described^27^. For crystallization, the wild-type enzyme was concentrated to 8–10 mg/ml and was placed in a solution of 50 mM glycyl-glycine pH 7.2, 50 mM KCl, 5% glycerol, by repeated rounds of centrifugal ultrafiltration at 4 °C.

The effect of NAG concentration on CPS1 activity was determined in an ornithine transcarbamylase-coupled assay. In this assay carbamoyl phosphate production was monitored colorimetrically as citrulline^27^ after 10-min incubation of the enzyme (wild-type or mutant form) at 37 °C. The assay mixture containing 50 mM glycyl-glycine pH 7.4, 70 mM KCl, 1 mM dithiothreitol, 20 mM MgSO_4_, 5 mM ATP, 35 mM NH_4_Cl, 50 mM KHCO_3_, 10 mM NAG, 5 mM L-ornithine, 4 U/ml of ornithine transcarbamoylase and variable concentrations (0–50 mM) of NAG (neutralized with KOH).

### Crystallization

Initial crystallization screenings at 21 °C used a HoneyBee X8 robot (Genomics Solutions), the sitting-drop vapor-diffusion method in 96-well MRC plates, different protein concentrations, and several commercial crystallization screen kits from Qiagen, Jena Bioscience and Hampton Research. The *apo* crystals were obtained using a 1/1 mixture of protein solution (8 mg/ml, containing 1 mM AMPPNP, 5 mM MgCl_2_, 1 mM NAG and 10 mM KCl) and of crystallization solution (20% w/v PEG 3350 and 0.2 M trisodium citrate). The cryobuffer used for crystal harvesting into liquid nitrogen was the crystallization solution enriched with 20% (w/v) PEG 400. The *ligand-bound* crystal was obtained at 4 °C by supplementing the protein with 20 mM ADP, 60 mM MgCl_2_, 10 mM NAG and 40 mM KCl, using as crystallization solution 0.4 M MgCl_2_ and 0.2 M ammonium tartrate, utilizing for harvesting into liquid nitrogen a cryoprotectant solution containing the mother liquor plus 10% (w/v) PEG 3350 and 30% glycerol.

### Data collection and processing

X-ray diffraction data of *apo* and *ligand-bound* crystals were collected at 100 K (Oxford Cryosystems) at 0.961 Å and 0.979 Å wavelengths, respectively. *Apo* crystals were analyzed in the ID14-4 beamline of the ESRF (Grenoble, France) using an ADSC Quantum Q325r detector. *Ligand-bound* CPS1 was analyzed at beamline XALOC (ALBA, Barcelona, Spain) using a Dectris PILATUS3 6 M detector. Datasets processed with XDS^56^, converted with POINTLESS^57^, were scaled with SCALA^57^ at resolutions of up to 2.4 Å and 2.6 Å for *apo* and *ligand-bound* crystals, respectively. The *apo* crystal belonged to space group P2_1_ (unit cell parameters: a = 99.3 Å, b = 133.5 Å, c = 142.9 Å and β = 102.5º) and contained two CPS1 subunits in the asymmetric unit. The crystal of *ligand-bound* CPS1 belonged to space group P1 (a = 78.92 Å, b = 98.56 Å, c = 214.89 Å, and α = 90.66°, β = 98.65°, γ = 90.08°) and contained four CPS1 subunits in the unit cell. Table 1 gives details and statistics of crystallographic data.

### Phasing, model building, and refinement

Molecular replacement was successful with data for the *apo* crystal reprocessed in space group P1, using PHASER^58^ and, as search models, the heterodimer of EcCPS (PDB 1JDB)^34^ and the C-terminal domain of human CPS1 (PDB 2YVQ). This initial solution consisted of four CPS subunits in the P1 unit cell presenting clear deviations from a P2_1_ packing. Model improvement was achieved by an iterative process of rigid body refinement of domains forming each CPS subunit, followed by restrained refinement, including non-crystallographic symmetry restraints, with REFMAC5^59^ and graphic model-rebuilding with Coot^60^. A perfect pseudo-merohedral twinning (-h, -k, -l) was detected and taken into account by REFMAC5. The four subunits contained in the P1 cell experienced relatively large reorganizations approaching, as refinement progressed, a packing corresponding to a P2_1_ space group. Therefore, when the *R*_*free*_ was below 26% the structure of one of the CPS subunits was used to obtain a solution by molecular replacement with the original P2_1_ data. Refinement was continued in this P2_1_ space group. Isotropic B factors and TLS were used in the last steps of refinement, with TLS groups chosen according to the previously determined domains^27^. All diffraction data were used throughout the refinement process except the 5% randomly selected data used to calculate the *R*_free_. Stereochemistry of the model was checked and improved by analysis with PDB_REDO^61^ and MolProbity^62^ (final score was 1.16 (100th percentile)).

*Ligand-bound* crystal data were initially integrated and scaled in space group P2_1_. Molecular replacement with PHASER^58^ using a monomer of the previously solved *apo* model provided a solution with acceptable electron density map. Positioning of domains was improved by rigid body refinement. Several fragments absent in the *apo* model, as well as some regions showing important structural differences, were manually rebuilt with Coot^60^, in accordance to the density map and partially using the EcCPS model from PDB 1JDB as reference. This model was subjected to restrained refinement using REFMAC5^59^. However, refinement became stuck at high *R/R*_*free*_ values. Detailed inspection of the data and analysis with SFCHECK and Xtriage from the PHENIX package^63^ showed that the crystal presented pseudosymmetry and the correct space group was P1. Therefore, diffraction data were reprocessed and scaled in P1, resulting in a solution with four CPS1 subunits in the unit cell. Restrained refinement, with twinning-correction only in the first steps, and automatically generated non-crystallographic symmetry restraints, was performed with REFMAC5^59^. Ligands were incorporated to the model with Coot^60^. B factors and TLS (1 group per monomer) were used in the last refinement steps. The nature of the ions was decided during refinement taking into account the size of the electron density, the geometry of the coordination sphere with, in particular, the coordination distances and the type of ligands, and the presence of the ion in the crystallization drop. In the case of Ni, it was not present but could have leached out from the Ni-affinity column used for enzyme purification. Thermal factors of most ions were similar to the ones of the neighbouring protein atoms, further supporting the nature of the ions assigned. Stereochemistry of the model was improved and checked as described for the *apo* model. The MolProbity score was 1.45 (100th percentile).

The spaces of the cavities corresponding to the ammonia and carbamate transport tunnels, were analyzed with MOLE^64^. HOLLOW^65^ was used to fill the channels with dummy atoms for representation. Graphical representations of the structures were generated with PyMOL (http://www.pymol.org/).

## Additional Information

**Accession codes:** Atomic coordinates and structure factors have been deposited in the Protein Data Bank under accession codes 5DOT and 5DOU.

**How to cite this article**: Cima, S. *et al.* Structure of human carbamoyl phosphate synthetase: deciphering the on/off switch of human ureagenesis. *Sci. Rep.* **5**, 16950; doi: 10.1038/srep16950 (2015).

## Supplementary Material

Supplementary Information

Supplementary Video 1

## Figures and Tables

**Table 1:** Data collection and refinement statistics.

	*apo* CPS1	*Ligand-bound* CPS1
**Data collection**		
Space group	P2_1_	P1
Cell dimensions		
a, b, c (Å)	99.3, 133.5, 142.9	78.9, 98.6, 214.9
α, β, γ (º)	90.0, 102.5, 90.0	90.7, 98.7, 90.1
Matthews Coefficient	2.81	2.51
Resolution (Å)	44.5-2.40 (2.53-2.40)*	40.0-2.60 (2.74-2.60)*
Total Reflections	475,967 (59,848)*	329,157 (40,514)*
Unique Reflections	136,595 (17,237)*	178,654 (24,542)*
*R*_*sym*_ (%)	11.4 (34.2)*	8.1 (32.1)*
*I*/σ_*I*_	4.8 (2.0)*	7.9 (2.1)*
Completeness (%)	96.3 (83.4)*	90.7 (84.9)*
Redundancy	3.5 (3.5)*	1.8 (1.7)*
CC1/2 (%)^66^	98.5 (87.4)*	98.5(51.6)*
**Refinement**		
Resolution (Å)	43.95-2.40	40.00-2.60
No. reflections	129,708	169,655
*R*_*work*_/*R*_*free*_ (%)	16.5/19.6	19.5/22.9
No. atoms or molecules:		
Protein (monomers)	2	4
Protein (atoms)	21,001	44,040
Ligands (molecules or ions)		
ADP	0	8
NAG	0	4
Phosphate	0	4
Ni/Mg/K/Cl	2/0/0/0	4/12/18/3
Water	819	640
Average *B*-factors		
Protein atoms	40.5	46.1
Ligands		
ADP	–	26.2
NAG	–	21.5
Phosphate	–	21.9
Ni/Mg/K/Cl	34.5/–/–/–	50.4/23.3/41.5/33.7
Water	35.5	23.4
Ramachandran plot^62^		
Favored (%)	96.6	96.9
Outliers (%)	0.41	0.18
R. m. s. deviations		
Bond lengths (Å)	0.009	0.011
Bond angles (º)	1.29	1.40

^*^Values in parenthesis correspond to the highest resolution shell.
