# Supplementary material for: Structure of human carbamoyl phosphate synthetase: deciphering the on/off switch of human ureagenesis
Source: Sci Rep. 2015 Nov 23;5:16950. doi: 10.1038/srep16950 (PMC4655335; doi:10.1038/srep16950)
Supplement: Supplementary Information [file srep16950-s1.pdf]

SUPPLEMENTARY INFORMATION

Structure of human carbamoyl phosphate synthetetase: deciphering the on/off switch of human ureagenesis

Sergio de Cima, Luis M. Polo, Carmen Díez-Fernández, Ana I. Martínez, Javier Cervera, Ignacio Fita & Vicente Rubio

This Supplementary Information file for article *"Structure of human carbamoyl phosphate synthetase: deciphering the on/off switch of human ureagenesis"* includes:

- 3 supplementary tables
- 5 supplementary figures
- The caption for 1 supplementary video.

References given are those in the reference list of the main text of the paper.

Supplementary Table 1. ***Domains rotations between apo and ligand-bound structures of human CPS1.***

Rotations (in °) of every domain with respect to the indicated reference domain were estimated by comparing the *apo* and the *ligand-bound* structures of human CPS1. They were calculated after superposition of the reference domain using least squares fit in Coot[60].

	Rotation of the domain relative to the reference domain (º)				
Reference Domain	**S2**	**L1**	**L2**	**L3**	**L4[a]**
**S1**	0.8	2.4	2.7	3.8	11.6
**S2**		3.1	3.1	3.8	11.1
**L1**			3.3	4.8	13.0
**L2**				2.2	11.4
**L3**					9.3

[a] The C-tail of the L4 domain (residues 1481-1500) was excluded from the calculations of rotations

Supplementary Table 2. ***Buried surface area in the interdomain contacts of the apo and ligand-bound human CPS1 structures.***

The buried surface (Å^2) per domain at a given interdomain interface in the *apo* and *ligand-bound* human CPS1 structures was computed with the PISA server (http://www.ebi.ac.uk/pdbe/pisa)[28]. Surface area at a given interface was calculated as half of the difference between the total accessible surface areas of isolated and interfacing structures. Values in the last row indicate the total accessible surface area of each isolated domain.

**Buried surface per domain in the indicated domain-domain interaction (Å^2)**								
**Domain**	**S1**	**S2**	**Linker**	**L1**	**L2**	**L3**	**L4**	**C-tail**
***Apo* form**								
**S1**	-	1,309	-	671	837	-	-	-
**S2**		-	640	615	174	-	-	-
**Linker**			-	364	-	-	-	-
**L1**				-	1,828	1,824	122	255
**L2**					-	888	-	-
**L3**						-	1,165	365
**Total surface (Å^2)**	8,021	9,570	2,418	15,165	10,396	17,140	7,366	1,638
***Ligand-bound* form**								
**S1**	-	1,315	-	673	816	-	-	-
**S2**		-	644	660	175	-	-	-
**Linker**			-	496	-	-	-	-
**L1**				-	2,088	2,345	140	241
**L2**					-	864	-	-
**L3**						-	985	316
**Total surface (Å^2)**	8,037	9,605	2,535	16,851	10,361	16,583	6,990	1,257

Supplementary Table 3. ***Synthetic oligonucleotides used for C-terminal deletions***

**Deletion**	**Direction**	**Sequence 5'-3'**[a]
**Δ5**	Forward	CCACTACAGGCAGTACAGT**TGA**GGAAAAGCAGC
**Δ5**	Reverse	GCTGCTTTTCC**TCA**ACTCTGCCTGTAGTGGAAAAG
**Δ7**	Forward	GTCTTTTCCACTACAGGCAG**TAA**AGTGCTGGAAAAG
**Δ7**	Reverse	GCTGCTTTTCCAGCACT**TTA**CTGCCTGTAGTGG
**Δ9**	Forward	GAGTCTTTTCCACTAC**TGA**CAGTACAGTGCTGGAAAAG
**Δ9**	Reverse	CCAGCACTGTACTG**TCA**GTAGTGGAAAAGACTCTTGG
**Δ10**	Forward	CCAAGAGTCTTTTCCAC**TAA**AGGCAGTACAGTGCTGG
**Δ10**	Reverse	GCCAGCACTGTACTGCCT**TTA**GTGGAAAAGACTCTTG
**Δ11**	Forward	CCAAGAGTCTTTTC**TAA**TACAGGCAGTACAGTGCTGG
**Δ11**	Reverse	GCCAGCACTGTACTGCCTGTA**TTA**GAAAAGACTCTTG

[a] The mutated codon is highlighted in bold type

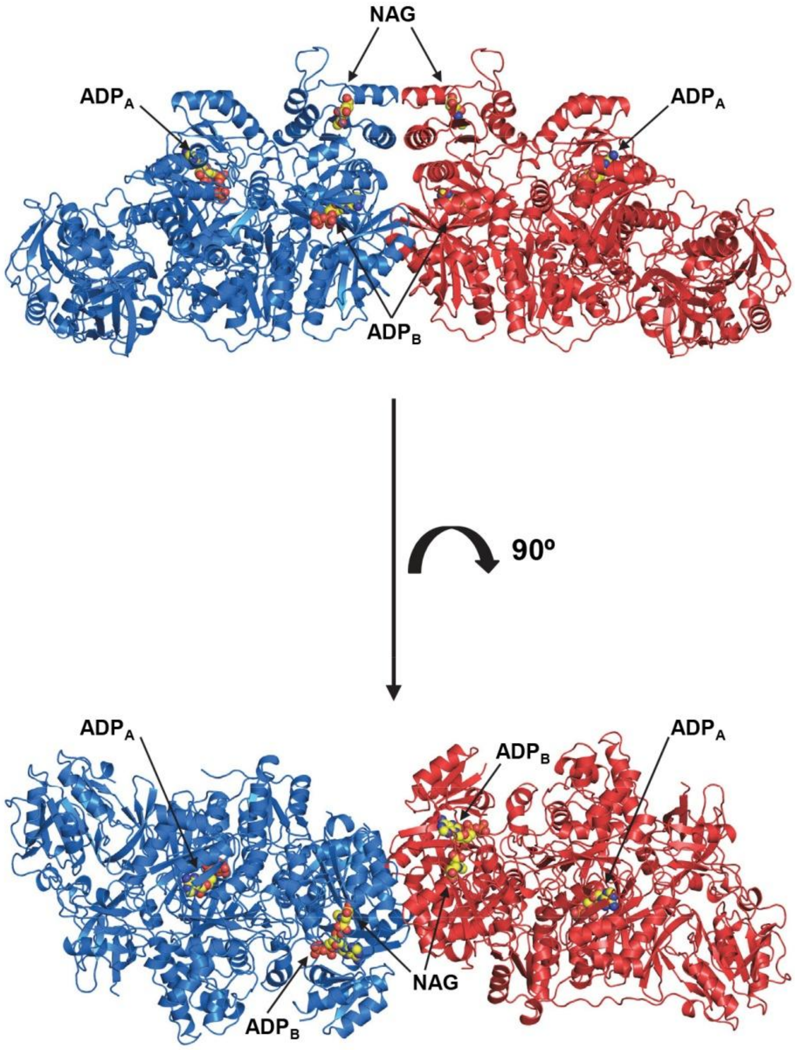

Supplementary Figure 1. ***Dimer of human CPS1.***

Two orthogonal views in cartoon representation of the dimer of the *ligand-bound* form of CPS1, with each monomer shown in a different color. Ligands (NAG and two ADP molecules per monomer) are shown in space-filling representation.

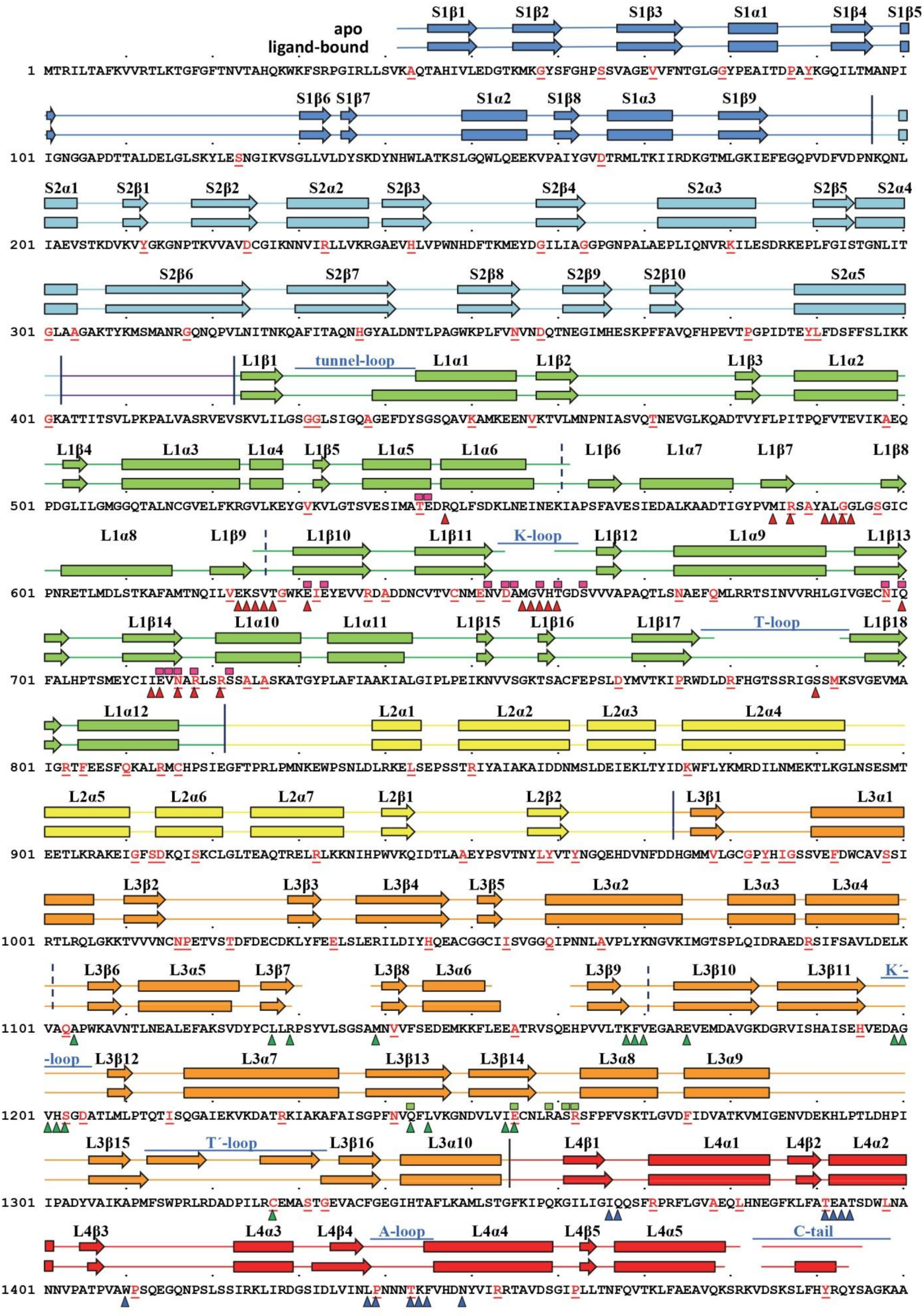

Supplementary Figure 2.

Supplementary Figure 2. ***Human CPS1 amino acid sequence and secondary structure assignment in the apo and ligand-bound structures.***

Amino acid sequence and secondary structure assignment (bars and arrows represent helices and beta strands respectively) for *apo (top)* and *ligand-bound (bottom)* structures. The nomenclature of helices and beta strands is based on the most complete structure, which is the *ligand-bound* one. Absence of continuous line on the secondary structure assignment indicates disordered regions that thus are not observed in the structural model. Loops showing important differences between both forms or being proposed as important (main text) in the activation mechanism are indicated in blue and named. Residues for which clinical mutations producing human CPS1 deficiency have been described[22] are colored red and are underlined. Triangles under the sequence denote residues involved in binding of ADP_A/Pi, ADP_B and NAG, (colored red, green or blue, respectively). Squares over the sequence indicate residues involved in binding of Mg^{2+}, K^+ or Cl^-, being colored red and green, respectively, when they are at the bicarbonate or carbamate phosphorylation sites. Solid vertical black lines mark interdomain boundaries. Broken vertical lines in domains L1 and L3 indicate the boundaries for the A, B and C subdomains of these two grasp domains.

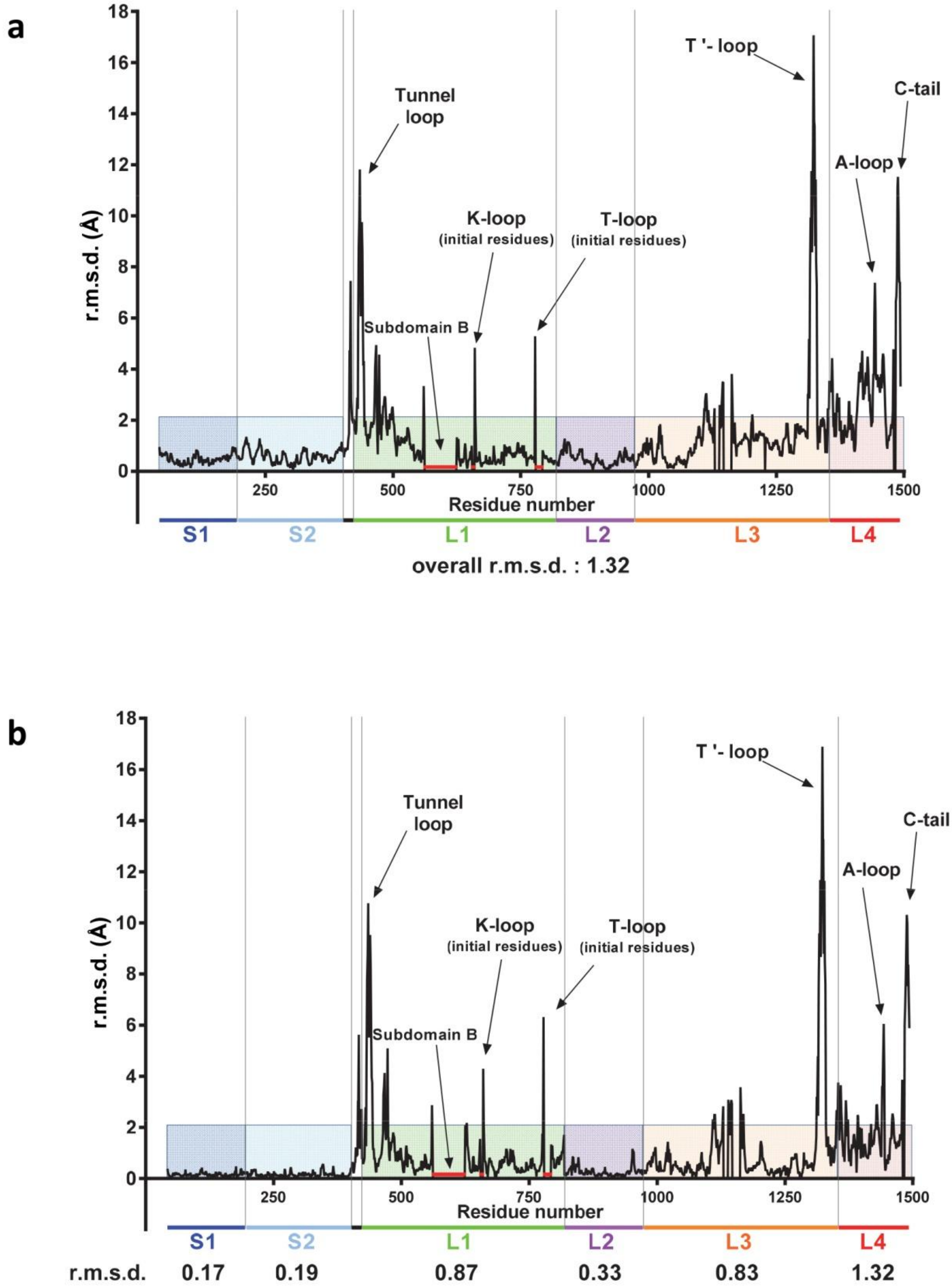

Supplementary Figure 3. ***Differences between apo and ligand-bound forms of CPS1.*** Graphic representation of the r.m.s.d. values for each residue from superimposition of the complete monomer **(a)** or of the individual isolated domains **(b)** of *apo* and *ligand-bound* forms of CPS1. Overall r.m.s.d. values for the whole monomer or the isolated domains are shown below the respective graph.

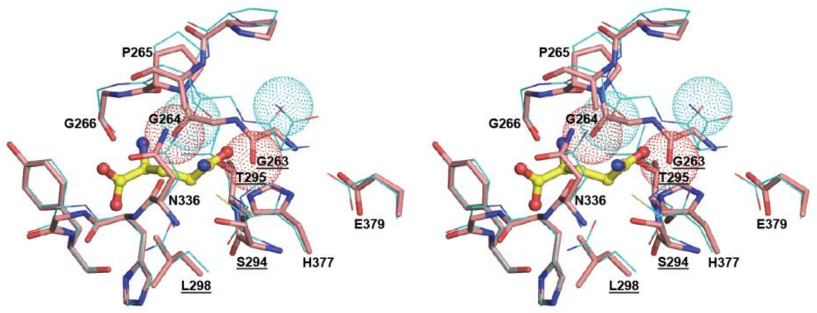

Supplementary Figure 4. ***The ancestral glutaminase site.***

Stereo view of the superimposition of the glutaminase site of EcCPS (shown as lines with C atoms colored cyan; PDB 1JDB) and the equivalent site of CPS1 (shown as sticks with C atoms colored salmon). The substrate glutamine has been modeled into EcCPS from the structure of the C269S mutant of EcCPS (PDB 1C3O). Some key residues of CPS1 are labeled; those underlined are not conserved with respect to EcCPS. The Van der Waals spheres of oxygen atoms of Gly263 and Gly264 of CPS1 and the equivalent residues of EcCPS are shown (red and cyan dotted spheres respectively) to highlight the steric clash of CPS1 with the modeled glutamine.

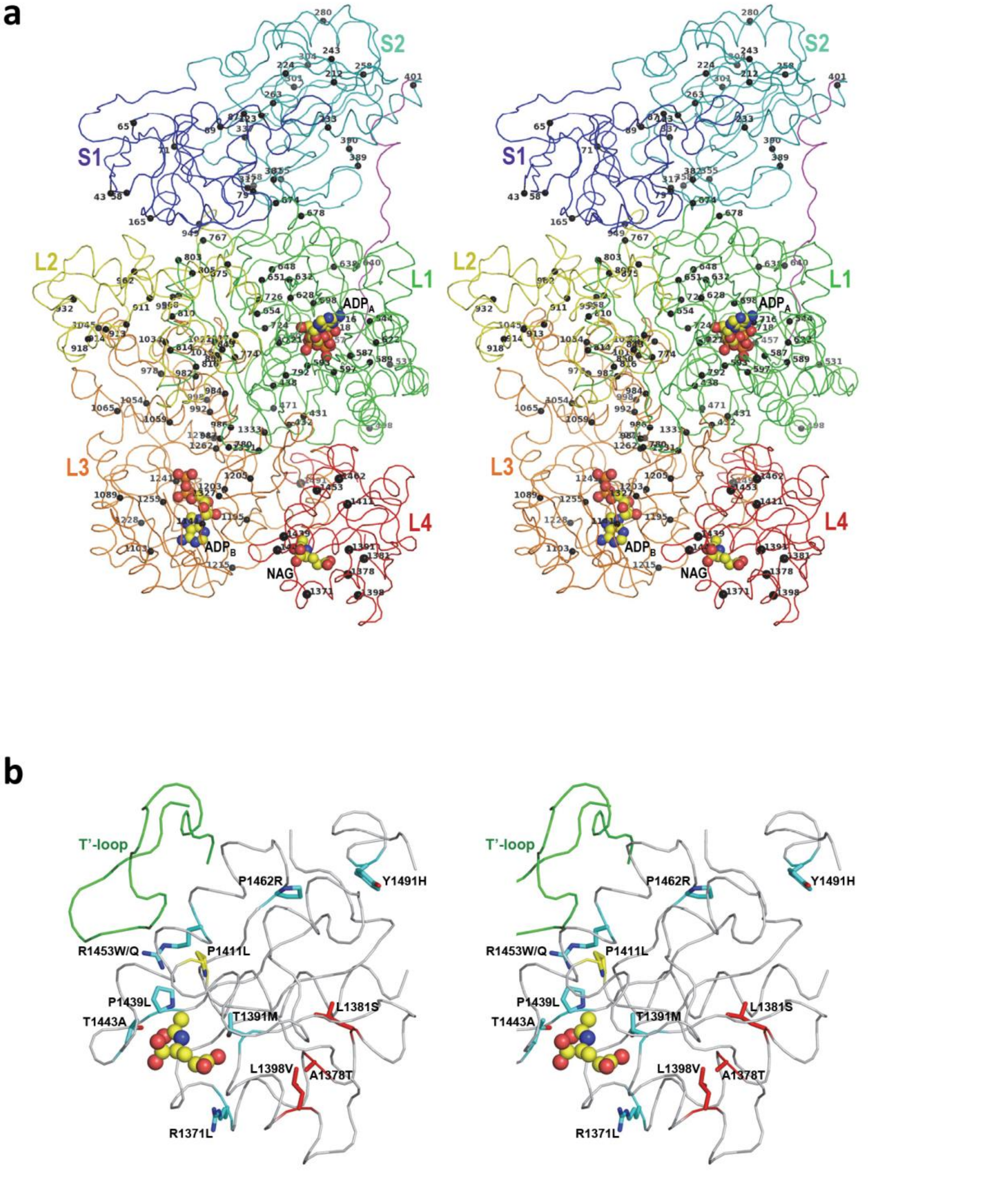

Supplementary Figure 5. ***Localization in the structure of human ligand-bound CPS1 of the residues for which missense mutations were reported in patients with CPS1 deficiency.***

(a) Stereo view of the entire enzyme monomer, localizing with spheres, and numbering them, the residues hosting missense mutations reported in CPS1-deficiency patients, highlighting in larger size the residues of the L4 domain, which are discussed here. The

ADP molecules and NAG are shown in space filling representation, and the different domains are labeled. **(b)** Close-up stereo view of the L4 domain labeling and showing the side-chains of the residues where clinical missense mutations have been reported[22,37]. Those in red have been associated with mutations destabilizing the enzyme, whereas the one in yellow with a mutation having minor effects. The T'-loop of the L3 domain is illustrated also (in green). Bound NAG is shown in space-filling representation.

Supplementary Video 1.

Conformational rearrangements on CPS1 upon NAG binding.

This movie shows a morph between the *apo* and *ligand-bound* forms of CPS1. First, *apo* CPS1 is shown with each domain in a different color. Next, a zoom view of the L4 domain shows the morph of this domain from the *apo* to the *ligand-bound* form, and reverse. NAG is progressively shown in spheres representation. The T´-loop is colored in magenta and some residues of the NAG binding site are shown in sticks representation to emphasize the conformational change. The next scene shows the same morph, from *apo* to the *ligand-bound* form and reverse, for the whole large moiety. The tunnel-loop and the T´-loop are colored magenta, and the L1 domain regions stabilized in the *ligand-bound* form (the K-loop, the T-loop and subdomain B) are shown in orange and appear gradually as the morph progresses. NAG and both ADPs are shown in spheres representation. In the final scene, the carbamate tunnel in the *apo* form (colored ochre) is shown and the view is reoriented to emphasize the cavities found in this form. The morph to the *ligand-bound* form is played again to show how the conformational changes lead to the formation of the functional carbamate tunnel (colored red). The morph was created with Morph2 (http://www.bioinformatics.org/pdbtools/morph2) and rendered with PyMOL using the eMovie plug-in (http://www.weizmann.ac.il/ISPC/eMovie.html) to compose the different scenes. This video can be reproduced best with VLC (www.videolan.org/vlc).
